# Dynamic Correlation between Intrahost HIV-1 Quasispecies Evolution and Disease Progression

**DOI:** 10.1371/journal.pcbi.1000240

**Published:** 2008-12-12

**Authors:** Ha Youn Lee, Alan S. Perelson, Su-Chan Park, Thomas Leitner

**Affiliations:** 1 Department of Biostatistics and Computational Biology, University of Rochester Medical Center, New York, United States of America; 2 Theoretical Biology and Biophysics, Los Alamos National Laboratory, Los Alamos, New Mexico, United States of America; 3 Institute of Theoretical Physics, Cologne University, Köln, Germany; Utrecht University, Netherlands

## Abstract

Quantifying the dynamics of intrahost HIV-1 sequence evolution is one means of uncovering information about the interaction between HIV-1 and the host immune system. In the chronic phase of infection, common dynamics of sequence divergence and diversity have been reported. We developed an HIV-1 sequence evolution model that simulated the effects of mutation and fitness of sequence variants. The amount of evolution was described by the distance from the founder strain, and fitness was described by the number of offspring a parent sequence produces. Analysis of the model suggested that the previously observed saturation of divergence and decrease of diversity in later stages of infection can be explained by a decrease in the proportion of offspring that are mutants as the distance from the founder strain increases rather than due to an increase of viral fitness. The prediction of the model was examined by performing phylogenetic analysis to estimate the change in the rate of evolution during infection. In agreement with our modeling, in 13 out of 15 patients (followed for 3–12 years) we found that the rate of intrahost HIV-1 evolution was not constant but rather slowed down at a rate correlated with the rate of CD4+ T-cell decline. The correlation between the dynamics of the evolutionary rate and the rate of CD4+ T-cell decline, coupled with our HIV-1 sequence evolution model, explains previously conflicting observations of the relationships between the rate of HIV-1 quasispecies evolution and disease progression.

## Author Summary

Saturation of sequence divergence and a decline of diversity in later stages of infection have been commonly observed during HIV-1 infection, although the length of the time to acquired immunodeficiency syndrome (AIDS) is highly variable among patients. To explain this common feature, we developed a simple sequence evolution model with two main components: (i) fitness, the number of offspring produced, and (ii) the proportion of offspring that are mutants. Assuming a decrease in the proportion of offspring that are mutants as virus variants evolve further from the founder strain, we were able to fit the universal trends of divergence and diversity. In contrast, neither the model with gradual increase of fitness nor the model with rapid emergence of virus variants with greater fitness explained the dynamics of divergence and diversity. The prediction of the model was confirmed in the majority of longitudinally followed patients; the rate of HIV-1 evolution was stationary before disease progresses; however, the rate slowed down at a rate correlated with the rate of immune cell decline. Deciphering dynamic correlation between the rate of HIV-1 evolution and the kinetics of immune cell level united previous conflicting observations of the relationships between the rate of HIV-1 evolution and disease progression.

## Introduction

Within an HIV-1 infected individual, the HIV-1 population evolves under host immune response selection pressures [1]–[3]. Development of genetic diversity within the host results from a high virus replication error frequency (3.4×10^−5^ mutations site^−1^generation^−1^ [4]) coupled with an *in vivo* virus production rate exceeding 10^10^ virions per day [5]. Both diversifying and purifying selection impact the evolution of HIV-1 sequences. In the absence of antiretroviral drug treatment, HIV-1 must balance the preservation of important life cycle functions with the ability to escape host immune surveillance.

The interaction between the HIV-1 population and the host is revealed in the following observations: First, an increase of fitness during the course of chronic infection has been demonstrated by comparing the replication rate of virus genomes isolated at early times following infection with that of later viruses [6]. Second, although CD8+ T-lymphocytes restrain virus replication in HIV-1 infection, escapes from both CD8+ T-cell responses and neutralizing antibodies are well documented [7]–[9]. Studies on CD8+ T-cell response to autologous virus Env, Gag, and Tat proteins observed variation at epitope-containing sites in the HIV-1 population [10],[11]. Such variation implies escape from CD8+ T-cell responses. Furthermore, changes in N-linked glycosylation sites in Env have been observed in viruses that escape antibody neutralization [12].

Two measures have been used to describe HIV-1 evolution quantitatively, *diversity*, the genetic variation at a given time, and *divergence*, the genetic distance to a reference point, usually the founder virus. While several studies have investigated these measures, a detailed study carried out by Shankarappa *et al.* followed 9 patients longitudinally over 10–15 years [13]. They found that in the first phase of the asymptomatic period, both viral divergence and diversity increased linearly in the C2-V5 region of *env*. In a second phase, the viral population continued to diverge from the founder strain at the same rate, while diversity started to plateau or constrict. In the final phase, divergence stabilized and diversity declined. The decline of diversity was associated with the emergence of viruses using the CXCR4 coreceptors, expressed on both memory and naive cells, more so on naive T cells [14]–[17]. The stabilization of nonsynonymous divergence was reported to be more pronounced than the synonymous divergence at the late stage of infection [18],[19], suggesting reduced immune selective pressure.

The rate of intrahost HIV-1 sequence evolution has been correlated with the progression of the disease, which shows a considerable variability among patients (from a few months to 20 or more years). Several studies have found an inverse relationship between the rate of viral diversification and the host disease progression rate [1], [2], [18], [20]–[22], while others have not [23],[24]. In addition, it has been suggested that the level of genetic diversity that can be controlled by the host immune system is limited, and that exceeding a diversity threshold may be a key factor for disease progression [25]. More recently, Lemey *et al.* found an association between the synonymous substitution rate of HIV-1 and disease progression parameters [19]. Subjects with moderate disease progression from Shankarappa *et al.* [13] displayed a faster rate of synonymous substitutions in comparison to subjects with slow disease progression. It was speculated that a longer viral generation time may be responsible for a slower rate of synonymous substitutions and slower disease progression.

To unify all these observations, i.e., the universal intrahost dynamics of divergence and diversity and the contradicting observations between the rate of disease progression and the rate of intrahost evolution, here we propose a simple sequence evolution model that includes a mutation probability and a fitness value of sequence variants. The model accurately described HIV-1 sequence evolution within a patient, reflecting the dynamics of divergence and diversity over the infection by suggesting a slowdown of the evolutionary rate as disease progresses. We then measured the dynamics of intrahost HIV-1 sequence evolution from 15 previously followed patients and linked the change in the evolutionary rate to the dynamics of the CD4+ T-cell count. Deciphering the dynamics of intrahost HIV-1 quasispecies evolution allowed us to explain previously reported contrasting relationships between the speed of HIV-1 quasispecies evolution and disease progression.

## Results

### Sequence Evolution Model

To interpret the common dynamics of the divergence and diversity within a host in the chronic phase of HIV-1 infection, we developed a sequence evolution model where each viral sequence is represented by its distance to the founder strain, *d*. In this model, the number of sequences, *N*(*d*,*t*), at a distance *d* from the founder strain at time *t* is dictated by two factors: 1) the fitness, *F*(*d*), defined as the total number of offspring sequences from sequence *d* generated per unit time; and 2) the probability, *M*(*d*), of sequence *d* to evolve to sequence *d*+1 per unit time. Here we assume that the unit of time is chosen such that the probability of evolving to distances greater than *d+1* in one time unit is negligible.

**Figure 1:**
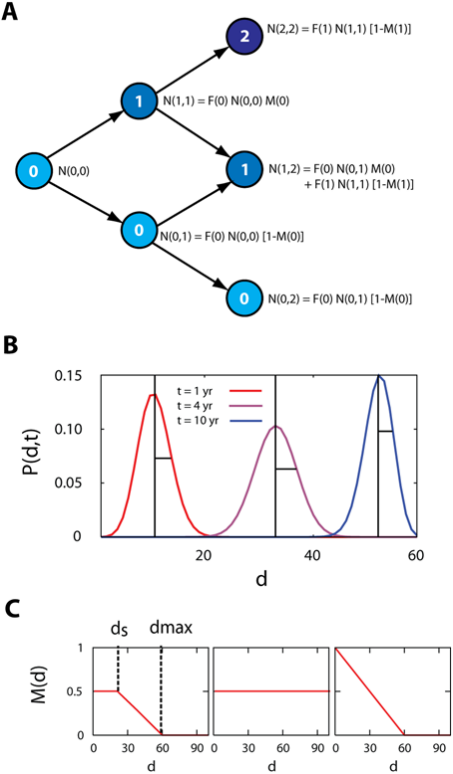
Schematic diagram of the HIV-1 sequence evolution model. (A) Each sequence is represented with a sequence index, *d* (the number within the circle), equal to the distance from the founder strain. *N*(*d*,*t*) denotes the total number of sequences at distance *d* at time *t*. *F*(*d*) is the total number of sequences produced per unit time per sequence. A sequence *d* at time *t* generates either sequence *d*+1 with probability *M*(*d*) by a mutation, or sequence *d* with probability 1−*M*(*d*) at time *t*+1. *M*(*d*) is the proportion of offspring that are mutants. (B) The divergence is defined as the mean and the diversity is defined as the standard deviation of the distribution of *P*(*d*,*t*) in Eq. (2). The position of the mean (divergence) is shown as the vertical line of each *P*(*d*,*t*) at year 1, 4, and 10, respectively. The standard deviation (diversity) is shown as the horizontal line of each distribution. (C) The profile of *M*(*d*) for the general (full) sequence evolution model (left panel), submodel 1 (middle panel), and submodel 2 (right panel). Here *d_s_* denotes the distance from which *M*(*d*) starts to decline and *d*_max_ denotes the distance point of *M*(*d*) = 0.

As shown in Figure 1A, at time 0, the total number of copies of virus 0 is *N*(0,0). At time 1, the total number of offspring sequences from virus 0 is *F*(0)*N*(0,0). Out of this total number of offspring, the number of mutant sequences is *F*(0)*N*(0,0)*M*(0), and the number of non-mutant sequences is *F*(0)*N*(0,0)(1−*M*(0)). Hence *M*(*d*) denotes the proportion of offspring that are mutants. In general form, this process is expressed as
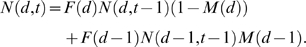
(1)


This model simulates the growth of the true genetic distance over time. Rather than a simple Hamming distance, which for finite sequence lengths cannot grow at a constant rate, the genetic distance we emulate is the distance realistic substitution models attempt to estimate [26]–[28]. In our later tree analyses of real data, we have used a general-time-reversible model that includes rate variation across sites that has been shown to realistically describe HIV-1 nucleotide evolution [26],[29]. We show below that the evolutionary rate of the 15 patients we analyze is approximately 10^−3^ per site per month. Since we analyze about a 600 nucleotide region of the HIV-1 env gene, this implies that we expect less than one substitution per month. Thus, a time unit of approximately one month is appropriate to analyze this data. Thus, our model is not following all the point mutations that can occur due to reverse transcription but rather simulates the growth of the true genetic distance from the founder in the presence of selection. In reality, multiple variants can exist at the same distance from the founder strain. In our model those variants have the same identification index, *d*, the distance from the founder strain and this implies that we need a measure of diversity that does not rely directly on sequence information but rather on the distribution of genetic distances *d*.

**Figure 2:**
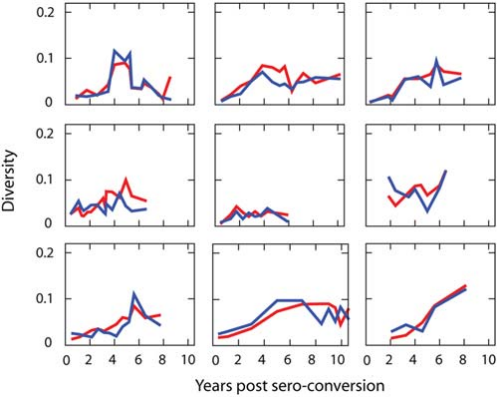
Two measures of diversity dynamics of 9 longitudinally followed patients. Diversity dynamics of each subject from the definition of average pairwise distance [red line] among all the sequences sampled at the same time point and the standard deviation [blue line] of the distribution of the tree distances of all the sequences at the same time point from the founder strain multiplied by a constant factor. The constant factors are 20, 20, 25, 25, 27, 40, 40, 30, 70 for S-P1 to S-P11, respectively. The two measures of diversity are proportional to each other.

Since an approximately constant number of sequences were sampled at all time points [13], we consider the normalized distribution of the distances at time *t*, i.e.,
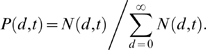
(2)The divergence *D_divergence_*(*t*), *i.e.*, the average number of nucleotide substitutions that accumulated along the branch from the founder strain as a function of time [13], is measured by the mean value of *d*, i.e.,
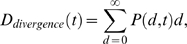
(3)(Figure 1B). The diversity *D_diversity_*(*t*), measured by Shankarappa *et al.* [13] as the average pairwise nucleic acid distance between all sequences at time *t*, is here measured with the standard deviation of *P*(*d*,*t*) as
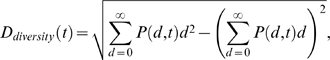
(4)(Figure 1B). In our model, because we do not discriminate between the variants at the same distance, we measure the level of diversity with the level of spread in the distance from the founder strain. This measure is an approximation made for consistency with our modeling approach. To examine this approximation, we calculated the measure of genetic diversity at the nucleotide level used by Shankarappa *et al.* and the measure of the standard deviation of the distance distribution from the founder strain, Eq. (4), for the same data in [13]. The two measures were found to be proportional to each other (Figure 2).

We assumed that the probability of mutation varied as a function of the distance from the original strain, *d*, according to *M*(*d*) = *m* if *d*≤*d_s_*, *m*(*d*_max_−*d*)/(*d*_max_−*d_s_*) if *d_s_*<*d*<*d*_max_, and *M*(*d*) = 0 if *d*≥*d*_max_, where *m* is a constant, *d_s_* is the starting point (distance) for the decline of *M*(*d*), and *d*_max_ is the distance at which *M*(*d*) = 0 (left panel in Figure 1C). The profile of *M*(*d*) directly reflects the retardation in the rate of sequence evolution as the virus evolves further from the founder strain.

**Figure 3:**
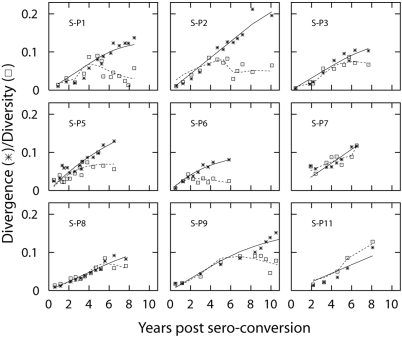
The fit of the full model to dynamics of divergence and diversity. Dynamics of divergence and diversity fitted with the full model (right panel in Figure 1C). We calculated divergence by first measuring the tree distance between a sequence sampled at time t and a strain found at the initial sample time point. Then we averaged all the pairwise tree distances between the sequences at t and the sequence sampled at the earliest time point. Likewise, the diversity was calculated from the data by averaging pairwise tree distances over all the sequences obtained at time t. We fixed parameters as *m* = 0.9 and *f* = 1 estimated *d_s_* and *d*_max_ using a non-linear least squares method based on the Levenberg-Marquardt algorithm [59] and calculated 95% C.I. of these estimated parameters based on bootstrap sampling of the residuals [60]. The result of the fit is summarized in Table 1.

To observe the effect of the profile of *M*(*d*) (left panel in Figure 1C) on the macroscopic evolution patterns, we first fixed the fitness as a constant, *F*(*d*) = *f*. Figure 3 shows the fit of the model to the dynamics of divergence and diversity of patients S-P1—S-P11 [13]. The fit of the model is summarized in Table 1. The method of calculating divergence and diversity dynamics is provided in Materials and Methods. Encouragingly, our model successfully quantified the dynamics of the divergence and the diversity based on first a constant evolutionary rate, then followed by a decline of the evolutionary rate (left panel in Figure 1C).

**Table 1:** Model fitting to divergence and diversity dynamics.

Subject	AIC_F_ (SQ_F_)	*d_s_*	*d*_max_	AIC_S1_ (SQ_S1_)	AIC_S2_ (SQ_S2_)
S-P1	−223.5 (6.78)	29.6 [26.7∶31.2]	46.2 [45∶47.6]	−215.4 (10.9)	−213.1 (10.8)
S-P2	−211.7 (5.16)	55.8 [53.0∶58.2]	61.1 [61.0∶61.2]	−206.0 (7.92)	−204.0 (7.75)
S-P3	−158.7 (1.44)	26.2 [24.1∶28.1]	64.1 [60.4∶67.1]	−154.3 (2.61)	−151.3 (2.62)
S-P5	−248.7 (5.47)	2.7 [2.7∶2.7]	58.6 [58.6∶58.6]	−249.0 (6.44)	−247.7 (6.19)
S-P6	−172.5 (0.67)	0 [0∶0]	24.8 [24.8∶24.8]	−165.9 (1.37)	−174.1 (0.74)
S-P7	−130.6 (2.21)	69.3 [69.3∶69.3]	95.0 [95.0∶95.0]	−134.5 (2.63)	−131.4 (2.63)
S-P8	−226.6 (1.25)	37.4 [37.4∶37.4]	62.3 [62.3∶62.3]	−224.2 (1.74)	−221.6 (1.74)
S-P9	−186.6 (2.85)	26.0 [24.2∶28.5]	66.2 [60.5∶72.9]	−188.2 (3.43)	−186.2 (3.33)
S-P11	−72.5 (1.43)	45.9 [45.9∶45.9]	97.6 [97.6∶97.6]	−69.9 (5.18)	−65.7 (5.18)

AIC is Akaike's information criterion with a second order correction for small sample sizes [61] and SQ is the sum of squared errors; subindex F indicates the full model; S1 submodel 1; and S2 submodel 2. The parameters *d_S_* and *d*_max_ are estimated from the full model (Figure 3C), along with 95% CIs (brackets) obtained by bootstrapping divergence and diversity dynamics 10^3^ times. The preferred model has the lowest AIC value.

### Special Cases of the Sequence Evolution Model

To further investigate the relationship between the profile of *M*(*d*) and the dynamics of divergence and diversity, we studied two special cases of *M*(*d*) in greater detail. Submodel 1 is defined by *M*(*d*) equal to a constant, *m* (middle panel in Figure 1C). For a constant fitness, *F*(*d*) = *f*, the normalized distribution of the distance at time *t*, *P*(*d*,*t*), satisfies
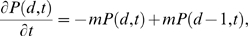
(5)with *P*(*d*,0) = 1 if *d* = 0 and *P*(*d*,0) = 0 otherwise. The generating function, 
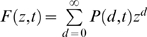
, satisfies the following equation,
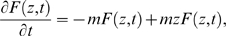
(6)with *F*(*z*,0) = 1. The solution of Eq. (6) is given by *F*(*z*,*t*) = e*^m(z−1)t^*. This implies that *P(d,t)* is a Poisson distribution, *P*(*d*,*t*) = *e* ^−*mt*^(*mt*)*^d^*/*d*!. The mean of this distribution gives the divergence, *D_divergence_*(*t*) = *mt*, and the standard deviation gives the diversity, 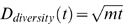. In this special case, both divergence and diversity increase as a function of time rather than saturate or decrease at later time points. Thus, assuming a constant fitness and constant evolutionary rate over the period of chronic infection fails to describe well the simultaneous intrahost dynamics of divergence and diversity. The fit of submodel 1 to the divergence and the diversity dynamics in all 9 patients is summarized in Table 1.

In submodel 2, we set *d_s_* = 0, resulting in a linear decrease of the probability of accumulated mutations per unit time, given by *M*(*d*) = (1−*d*/*d*_max_) for *d*≤*d*_max_ and *M*(*d*) = 0 for *d*>*d*_max_ (right panel in Figure 1C). In this case, from Eqs. (1) and (2), the dynamics of the evolution is summarized as
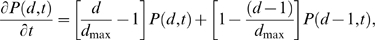
(7)for *d*≤*d*_max_ and ∂*P*(*d*,*t*)/∂*t* = 0 for *d*>*d*_max_.

Now the generating function 
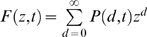
 satisfies
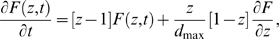
(8)with *F*(*z*,0) = 1 from *P*(*d*,0) = 1 when *d* = 0 and *P*(*d*,0) = 0 otherwise.

This equation can be solved using the method of characteristics. Let *z* and *t* be the functions of *s*. Then *F*(*z*,*t*) = *F*(*z*(*s*),*t*(*s*)) = *F*(*s*) and
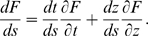
(9)If we choose the characteristic curve such that
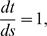
(10)with *t*(*s* = 0) = 0, we have *t* = *s*. By comparing Eq. (8) with (9), we obtain
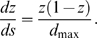
(11)By integrating Eq. (11), we have
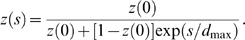
(12)Along this characteristic curve, by inserting Eqs. (8), (10) and (11) into Eq. (9), we obtain
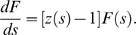
(13)By integrating Eq. (13), we obtain
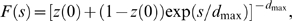
(14)where we have used the initial condition of *F*(*z*,0) = 1.

Since *s* = *t* and from Eq. (12),
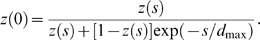
(15)If we insert Eq. (15) into Eq. (14), we obtain the solution of Eq. (8), 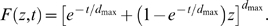
(16)Then *P*(*d*,*t*), the coefficient of *z^d^* of Eq. (16), is given as a binofmial distribution, 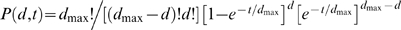. Hence, the divergence as a function of time is measured by the mean of this binomial distribution, 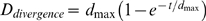, and the diversity is given by the standard deviation of *P*(*d*,*t*), 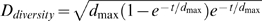. In submodel 2, the divergence first grows linearly and then saturates, and the diversity first increases and later decreases, which captures the saturation of divergence and the decline of the diversity at later stages of HIV-1 infection. The fit of submodel 2 to the divergence and the diversity dynamics of all 9 subjects is summarized in Table 1.

### Model Comparison

Comparing the full model and the two special cases using Akaike's information criterion (AIC) showed that in all the patients the full model fitted best except for S-P5, S-P7, and S-P9 (Table 1). Submodels 1 and 2 are interesting to consider because they are simpler and have analytical solutions. Comparing the two submodels to each other showed smaller or equal sum of squared errors for submodel 2 in all subjects except S-P3 (Table 1). One extra parameter in submodel 2, however, resulted in larger AIC values than for submodel 1 in all subjects except S-P6. Despite this, we prefer submodel 2 because it qualitatively captures the decrease of the diversity at the later stage of HIV-1 infection.

### Viral Fitness Effects

**Figure 4:**
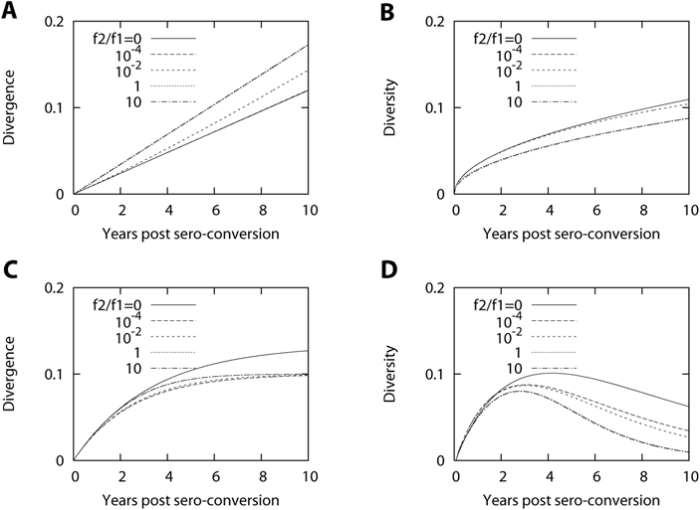
Dynamics of divergence and diversity with linear increase of fitness profile. Divergence (A) and diversity (B) as a function of time for *f*(*d*) = *f*_1_+*f*_2_ *d* and *M*(*d*) = *m* [submodel 1] for different values of *f*_2_/*f*_1_. The value of *m* is chosen as 0.5 and *f*_1_ = 1. The scale factors 300 and 20 for the divergence and the diversity are introduced to make comparable to the absolute values of measured divergence and diversity. Divergence (C) and diversity (D) as a function of time for *f*(*d*) = *f*_1_+*f*_2_ *d* and *M*(*d*) = 1−*d*/*d*_max_ for *d*≤*d*_max_ and *M*(*d*) = 0 for *d*>*d*_max_ [submodel 2]. The value of *d*_max_ is 40 and *f*_1_ = 1. The scale factor for the divergence is 500 and that for the diversity is 50.

We next studied the impact of viral fitness on the dynamics of the divergence and diversity. Recent *ex vivo* experimental data have suggested that the replication rate of viruses sampled at a later stage of HIV-1 infection is greater than that of viruses at an early stage of infection [6]. Therefore, we tested how fitness would affect the viral evolutionary pattern at the sequence level. First, we let fitness grow linearly as a function of the distance from the founder strain, *i.e.*, *F*(*d*) = *f*_1_+*f*_2_ *d*, in both submodels 1 and 2. In the range of *f*_2_/*f*_1_ from 0 to 10, we do not find any qualitative change in the patterns of divergence and diversity with time in either submodels 1 or 2 (Figure 4). This showed that an overall increase in fitness over the disease progression did not have a large effect on the diversity and divergence dynamics.

**Figure 5:**
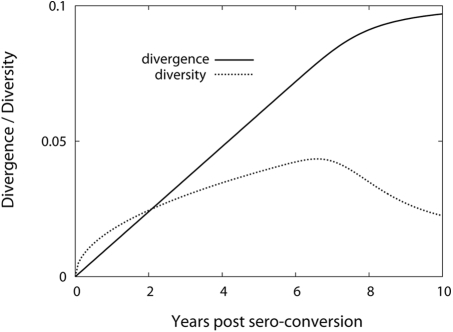
Dynamics of divergence and diversity with fitness reduction. Dynamics of divergence and diversity when fitness is reduced to 50% of its original value for *d*>*d_c_* = 50 mutations. For *d*≤50, *f* = 1 and for *d*>50, *f* = 0.5, and *M*(*d*) = 0.5 for all *d*. The saturation of divergence and the decrease of diversity are observed.

Second, we studied the case where the fitness is reduced after a given distance, *F*(*d*) = *f* for *d*≤*d_c_* and *F*(*d*) = *f*′ for *d*>*d_c_* where *f*′ is less than *f*. Here the proportion of offsprings that are mutants is constant for all viruses, *M*(*d*) = 0.5. We found that reduced fitness for viruses with a distance greater than *d_c_* reproduced the observed patterns for divergence and diversity. Figure 5 displays the calculated dynamics of divergence and diversity when we reduce the fitness of viruses having a distance greater than 50 mutations to 50% of the fitness of viruses with a distance less than 50 mutations. Although the profile of reduced fitness for the viruses after a given distance qualitatively explain the common dynamics of divergence and diversity, the reduction in the fitness of a virus population at later stages does not seem realistic considering the observation of increased fitness over the course of infection [6].

Finally, we investigated the case where only certain types of viruses may evolve to have a greater level of fitness. This situation has been described for emerging CXCR4-using viruses later in disease progression, and was found to correlate with a decline of diversity [13]. To simulate the outcome of emerging CXCR4-using viruses, potentially with greater level of fitness since they have a greater target cell range than CCR5-using viruses by infecting naïve CD4+ T-cells, we assigned a greater level of fitness to a fraction, α, of viruses having a distance larger than a critical value *d_c_*. This process is expressed as
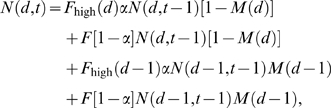
(17)where *F*_high_(*d*) = *F*_high_ for *d*≥*d_c_* and *F_high_*(*d*) = *F* otherwise. In this way, a proportion of viruses, 1−*α*, have fitness *F* and a proportion *α* have fitness *F*_high_ when *d*>*d_c_*. When d≤*d_c_*, the fitness is given by a constant *F*. We here chose *d_c_* = 50 mutations for the following reason. As we will show below, the average overall evolutionary rate for the 15 studied patients was estimated at approximately 10^−3^ nucleotide substitutions per site per month. This corresponds to 0.012 substitutions per site per year. With around 600 nucleotides in the dataset [13], 50 mutations corresponds to the mutations one expects to accumulate during ∼7 years. Thus, we chose the emergence of CXCR4-using viruses at *d* = 50 from this calibration since usually X4 viruses appear at later stages of infection.

**Figure 6:**
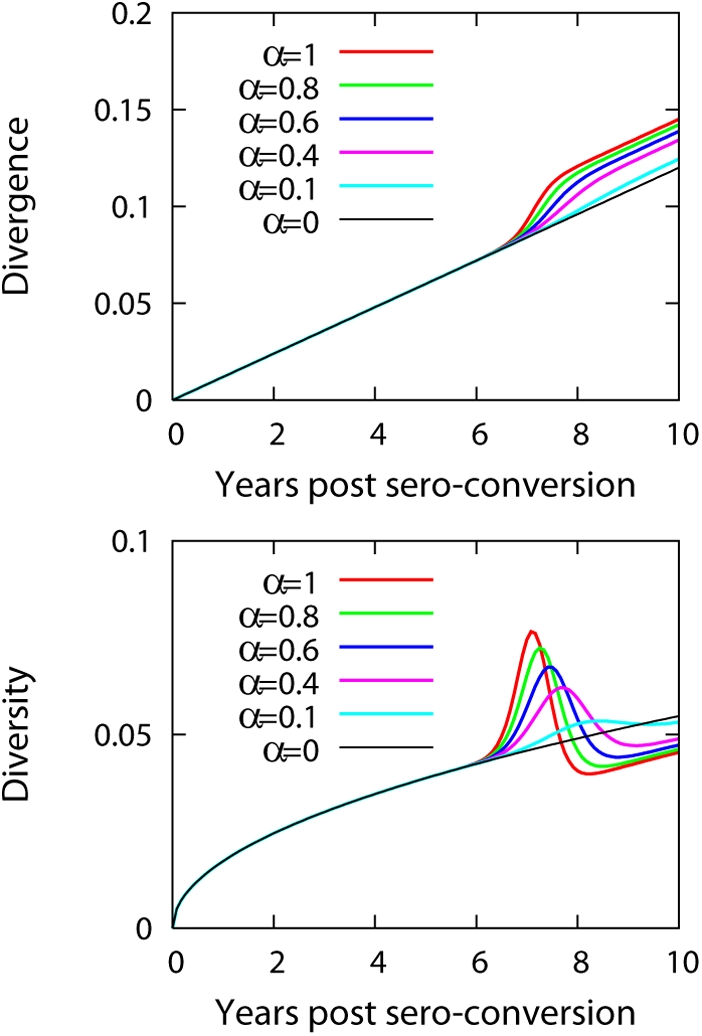
Dynamics of divergence and diversity with emergence of X4 viruses. Dynamics of divergence and diversity when imposing a greater level of fitness for certain types of viruses which emerge and persist, for example, by acquiring X4 tropism. The X4 viruses appear at *d* = 50 with greater level of fitness *F*_high_ = 1.5 in comparison to R5 viruses with fitness *F* = 1. The fraction of X4 viruses out of the total virus population with *d*≥50 is given by *α*. The rapid transient increases both in divergence and diversity upon the emergence of X4 viruses are observed. The scale factor for the divergence is 500, that for the diversity is 100, and *M*(*d*) = 0.5 for all *d*.

Figure 6 plots the dynamics of divergence and diversity by changing the fraction (*α*) of X4 viruses that have a 50% increase of fitness at distance *d_c_* = 50 mutations. As we increase the value of *α*, we observe an increase in divergence, then a transient rapid increase followed again by the inital slope of linear increase. The emergence and persistency of X4 viruses in the population leads to a rapid increase of diversity followed by a decline of diversity. Then at the final stage, diversity starts to increase again. This trend is robust to both the amount of fitness increase and the value of *d_c_*. For example, when we chose *d_c_* = 30, the transient rapid increases in the divergence and diversity still occur, but were shifted to 4.2 years. An initial rapid increase both in diversity and divergence due to the emergence of more fit virus is not compatible with the in vivo measurements from HIV-1 infected patients (Figure 3).

Overall, these simulations suggest that the probability profile of the evolutionary rate, *M*(*d*), rather than the fitness profile, *F*(*d*), is the main component in our model that determines realistic within-patient HIV-1 evolution.

### The Rate of Intrapatient Evolution Slows Down over the Infection

**Figure 7:**
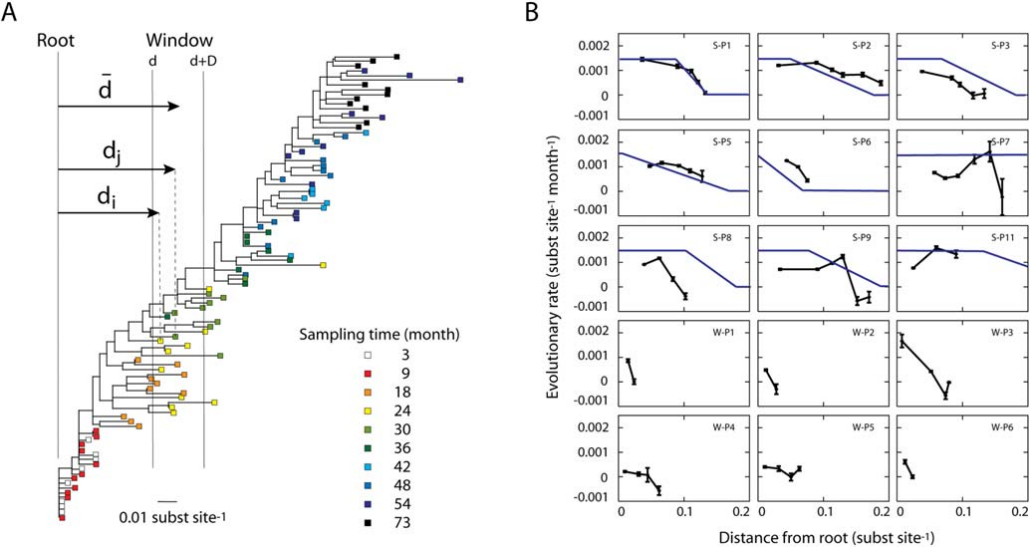
Evolutionary rate as a function of the distance from the root of the maximum likelihood tree of each patient. (A) Maximum likelihood tree for the viral sequences sampled from patient S-P6 over 6 years [13]. (B) Evolutionary rate as a function of the distance from the root of the tree for 9 patients from Ref. [13] and 6 patients from Ref. [22] (black lines). The evolutionary rate between sequence *i* and *j* is estimated by the distance difference, *d_j_*−*d_i_*, divided by the sampling time difference, *t_j_*−*t_i_*. The evolutionary rate at a certain distance from the root *d* was averaged over all possible sequence pairs (*i*, *j*) within a sliding window. The distance from the root for a particular window *d̅* is the average distance for all the sequences within that window. The size of the window (Δ) was 0.09 substitutions per site for S-P1 to S-P11 and 0.02 for W-P1 to W-P6. Error bars indicate ±1 standard deviation. The fitted rate of evolution with the full model to the divergence and diversity dynamics of each patient is depicted as blue line.

To test the prediction made by the model, i.e., a slowdown of the evolutionary rate as virus population evolves further from the founder strain, we calculated the rate of HIV-1 sequence evolution in consecutive windows over a maximum likelihood (ML) tree from each patient, starting from the root (see Materials and Methods). We used longitudinal sequence samples for 15 patients from two independent studies [13],[22]. As an example, Figure 7A shows the tree describing the HIV-1 evolution in patient S-P6. Figure 7B shows the resulting evolutionary rate as a function of the distance from the root for all patients. Interestingly, the rate is not constant but rather displays a dynamic behavior as HIV-1 evolves. In agreement with our model predictions, 13 out of the 15 patients showed a decline of the evolutionary rate as the sequence population evolved further from its founder strain. The same dynamic behavior was observed using other window sizes (Δ = 0.06 for the Shankarappa data and Δ = 0.03 for the Wolinsky data). Thus, the observed decline of the evolutionary rate was robust to the size of the window. In Figure 7B, we also plotted the evolutionary profile obtained by a fit to the divergence and diversity dynamics with the full model. The dynamics of the evolutionary rate calculated from the maximum likelihood tree was reasonably consistent with that obtained by a model fit to the divergence and diversity dynamics for each patient.

Sometimes we observed negative evolutionary rates in some patients when the distance from the root was large, mostly in later stages of infection (Figure 7B) when the sequence population hardly evolves any more. As a consequence some sequence variants may have a smaller distance from the founder stochastically, and if enough of such variants are detected then a negative evolutionary rate will be apparent. Also, the apparent negative rate of evolution may be due to the emergence of less evolved strains from latent reservoirs at later sampling time points.

### The Rate of Evolution Correlates with CD4+ Count

**Figure 8:**
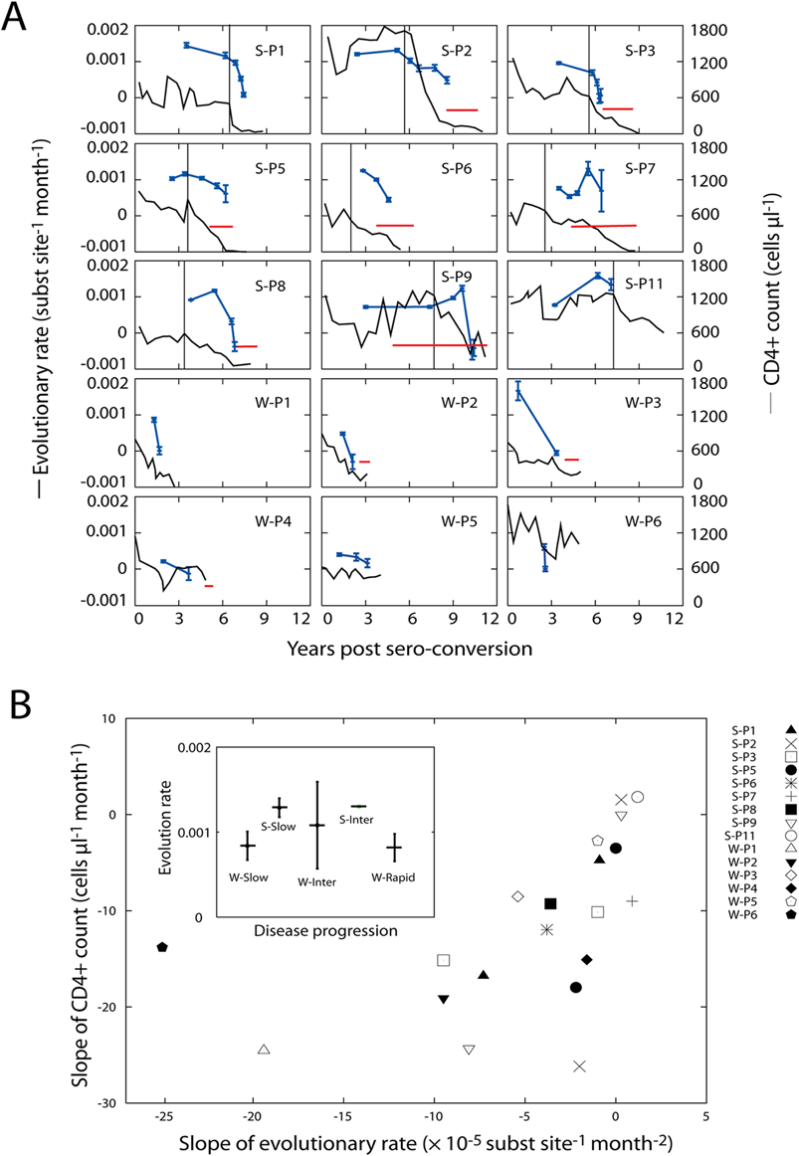
Dynamic correlation between the rate of HIV-1 evolution and the rate of CD4+ T cell count decline. (A) Evolutionary rate and CD4+ T-cell level as a function of time relative to seroconversion. Based on the estimation of the evolutionary rate as a function of distance to the root (Figure 1A), the evolutionary rate is plotted as a function of time (average sampled time point of all the sequences within the window). Error bars indicate ±1 standard deviation. The dynamics of the evolutionary rate is linked to that of the CD4+ T-cell count: While the CD4+ T-cell level is stable, the evolutionary rate is stable or increasing; the evolutionary rate starts to decrease when the CD4+ T-cell population is depleted. In patients S-P1 to S-P11, the dashed line indicates the stage when stable CD4+ T-cell count starts to decline. CD4+ T-cell counts were provided by J. Mullins and J. Learn. Red horizontal line denotes the period of antiretroviral therapy for each patient. (B) Correlation between the slope of CD4+ T cell count and the slope of the evolutionary rate (r = 0.68, P = 0.0014). For patients S-P1 to S-P11, the slopes are calculated separately before and after the dashed line. For W-P1 to W-P6, the slopes are measured over the whole range of the data. Note that the slope of the evolutionary rate for W-P6 is very large due to tight sampling, and the slope of the CD4+ T cell count is also high in the corresponding time interval, leading to W-P6 becoming an outlier. The inset shows the average evolutionary rate for different rates of disease progression. Each subject's average evolutionary rate is measured as the ratio between the root distance difference and the sampling time difference, averaged over all the sequence pairs in each tree. The error bars indicate ±1 standard deviation. Because we rooted our trees using a sequence from the initial time point, and not the clade B consensus as done by Wolinsky *et al.* [22], our calculated evolutionary rate differs from theirs. Subjects S-P2, S-P3, S-P7, S-P9, S-P11, W-P5, and W-P6 were classified as slow disease progressors; S-P1, S-P5, S-P6, S-P7, S-P8, W-P3, and W-P4 as intermediate progessors; and W-P1 and W-P2 as rapid progressors.

When the rate of change of the evolutionary rate was compared to the rate of change of CD4+ T-cell counts (Figure 8A), a significant correlation (r = 0.68, P = 0.0014) was observed (Figure 8B). In the initial interval where CD4+ T-cell counts were relatively stable (to the left of the dashed bar in Figure 8A), the evolutionary rate stayed relatively stable too. As CD4+ T-cell counts decreased and disease progressed in the patients the evolutionary rate slowed down. However, if one compares the overall (average) evolutionary rate from the whole study period (as defined by Eq. (20) in Materials and Methods), not its slope, with the disease progression rate, no clear correlation was seen (Figure 8B inset). The overall evolutionary rate of 15 patients was 10.4±3.14×10^−4^ substitutions per site per month. Note that increased or stable viral RNA counts rather than contraction in viral loads were observed in 7 patients under antiretroviral therapy in [13]. Thus, the decrease in the rate of evolution seems not to be associated with the onset of therapy.

**Figure 9:**
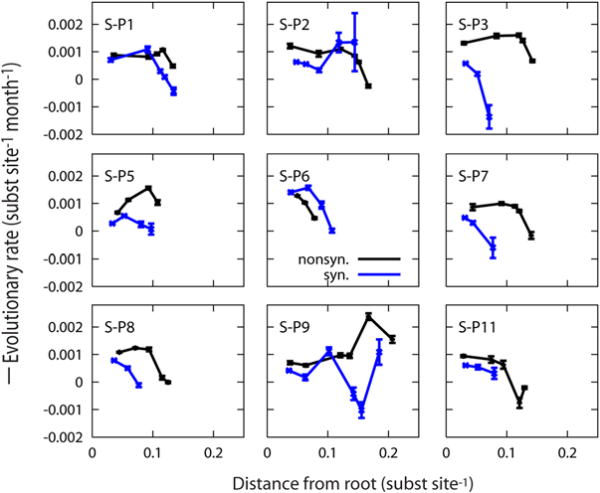
Dynamics of synonymous and nonsynonymous evolutionary rates. Synonymous (blue lines) and nonsynonymous (black lines) evolutionary rates as a function of the distance from the root of the tree for 9 patients from Ref. [13]. Synonymous and nonsynonymous rates were calculated using maximum likelihood trees based on only synonymous and non-synonymous substitutions, respectively, which were inferred using HyPhy with optimized MG94xREV models [30].

We estimated overall synonymous and nonsynonymous evolutionary rates across maximum likelihood trees based on synonymous and nonsynonymous changes only using HyPhy [30]. Similar to the overall total substitution rate, we found that neither synonymous nor nonsynonymous overall evolutionary rates correlated with the disease progression rate. For progressors with progression time less than seven years (S-P1, S-P5, S-P6, S-P7, and S-P8), the average synonymous and nonsynonymous evolutionary rates were estimated at 6.6±3.5×10^−4^ and 12±5×10^−4^ substitutions per site per month, respectively. For slow disease progressors with progression time greater than seven years (S-P2, S-P3, S-P7, S-P9 and SP-11), the average synonymous and nonsynonymous evolutionary rates were estimated at 6.8±2.3×10^−4^ and 13±4.5×10^−4^ substitutions per site per month, respectively. Lemey *et al.* reported lower overall synonymous evolutionary rates for these same slow disease progressors [19]. These contradictory observations may be explained by the use of different methods in the estimation of the overall evolutionary rates. While Lemey *et al.* used codon substitution models with a Bayesian relaxed clock model, we estimated the overall synonymous and nonsynonymous evolutionary rates in separate maximum likelihood trees based on synonymous and nonsynonymous changes [30] to allow for detecting rate changes across the trees. A common finding with Lemey *et al.* is that they also reported higher nonsynonymous rates (8.2±3.0×10^−4^) than synonymous rates (3.8±1.9×10^−4^). Importantly, although the overall synonymous evolutionary rate did not correlate with the disease progression rate in our calculations, we found that both synonymous and nonsynonymous evolutionary rates decline as disease progresses in 7 and 8 out of 9 patients in Ref. [13], respectively (Figure 9).

### Recombination Had a Minor Effect on the Evolutionary Rate

It is well known that HIV-1 recombines during its evolution. Therefore, we investigated whether recombination could have obscured our estimates of the evolutionary rates. All patient populations showed some signal for recombination (Table 2). This signal was, however, strongly correlated to the degree of homoplasy (r = 0.91). The homoplasy also grew with number of sequences per patient (R = 0.92), and all patients showed departures from neutral evolution, suggesting stochastic effects as well as selective environments rather than recombination. Most importantly, all our ML trees showed a clear time order of how sequences had been sampled through time (Figure 6), and additional trees calculated using SplitsTree showed that if recombination had occurred, then mostly samples taken close in time had been involved (data not shown). Thus, although difficult to exactly quantify, recombination had no large effect on our estimates of the evolutionary rate.

**Table 2:** Polymorphism and population recombination parameters of the studied sequence data.

Sequence Set	Number of Sequences	SITES gamma	SITES c/u	SITES Hud4Nc	PAUP HI	SNAP ds/dn	Tajima's D
S-P1	137	41.494	0.8835	40.016	0.596	1.52	−1.0657
S-P2	132	60.153	1.2432	55.695	0.641	0.8	−1.1133
S-P3	106	52.052	1.4195	35.629	0.552	0.93	−1.0346
S-P5	160	60.342	1.3264	58.403	0.618	1.34	−1.4946
S-P6	98	42.382	1.2009	64.24	0.521	1.49	−1.5263
S-P7	107	54.818	1.2341	62.637	0.605	0.83	−1.0209
S-P8	119	41.083	0.8691	66.185	0.555	1.28	−1.4212
S-P9	121	41.108	0.8424	42.343	0.624	0.89	−0.9025
S-P11	52	44.522	1.1115	10.185	0.443	1.88	−1.6284
W-P1	42	32.392	1.124	365.181	0.287	1.98	−2.1969
W-P2	44	23.687	0.7155	71.602	0.222	1.21	−2.3736
W-P3	35	19.477	0.7567	28	0.183	0.76	−0.1127
W-P4	58	28.297	0.6549	18.005	0.347	1.2	−1.9039
W-P5	70	31.498	0.6599	30.479	0.416	0.97	−2.0245
W-P6	39	11.117	0.7461	49.973	0.177	2.04	−1.0941

SITES gamma is a recombination rate estimate based on [46], and SITES Hud4Nc is based on [62]. SITES c/u is a ratio of the number of recombination events per mutation, *i.e.*, gamma divided by Theta (4Nu). PAUP HI is the homoplasy index calculated using PAUP* [58]. SNAP ds/dn is the average synonymous/non-synonymous ratio per patient population calculated using SNAP [41]. Tajima's D is a measure of departure from a neutral Fisher-Wright model [48].

## Discussion

The objective of this study was to develop a sequence evolution model and use it to investigate the relationship between nucleotide substitutions and disease progression within HIV-1 infected patients. In particular, we focused on explaining the pattern in which divergence from the founder increases linearly with time since infection and then saturates, whereas sequence diversity increases and ultimately declines. With these aims we developed a sequence evolution model, fitted the model to the divergence and diversity dynamics, and investigated two previously described datasets with rich HIV-1 nucleotide sequence data and CD4+ T-cell counts over time. Two important conclusions could be drawn from this study. First, we found that a model in which the survival of HIV-1 mutants was dictated by the distance from the founder strain accurately simulated HIV-1 within-patient evolution. This model could realistically simulate previously observed patterns of HIV-1 nucleotide sequence diversity and divergence over time by introducing an initially constant evolutionary rate later followed by a decline of the rate. Second, the evolutionary rate of HIV-1 within a patient follows the decline of the CD4+ T-cell count over time. Thus, the evolutionary rate of HIV-1 is not constant over time, but rather evolves in a dynamic way. This dynamic feature provides an explanation for previously conflicting observations of the relationship between the rate of HIV-1 quasispecies evolution and disease progression.

Three factors may contribute to the decrease of HIV-1's evolutionary rate as a function of disease progression. First, a decrease in the number of target cells of HIV-1 may increase the effective viral generation time. At the late stage of infection, the overall CD4+ T-cell count drops rapidly while viral load increases [13]. Lymph node immunohistologic alterations in HIV-1 patients as well as progression to a burned-out lymph node accompanying end-point lymphocyte depletion in SIV have been reported [31],[32]. Rapid loss of CD4+ T-cells in parallel with viral load increase might suggest that the proportion of infected cells out of total CD4+ T cell population is escalated as disease progresses. Our observation of a positive correlation between the slope of the evolutionary rate decrease and the slope of the CD4+ T cell count decline supports this view (Figure 8). Furthermore, the dynamics of the synonymous substitution rate shows qualitatively a similar pattern as the dynamics of the total evolutionary rate. Thus it follows that a decrease of the synonymous rate in most patients suggests an elongation of the effective viral generation time. In agreement, it was recently suggested that a slower rate of synonymous substitutions in patients with slower progression to AIDS was indicative of longer viral generation times [19]. Second, a weakening of immune selection pressure, as measured by the CD4+ T-cell count, may lower the observed evolutionary rate (Figure 8). Calculating the evolutionary rate in windows across a tree allowed us to detect a clear correlation between the slope of the evolutionary rate and the slope of the CD4+ T-cell count. Hence, deceleration of HIV-1 sequence evolution occurs in response to decreased immune selection. Not surprisingly, HIV-1 intrahost sequence evolution follows a principle of quantitative genetics where the response to selection is directly proportional to the intensity of selection [33]. If this scenario is operating, then the nonsynonymous evolutionary rate should decrease with disease progression. Here, we found that both the nonsynonymous and synonymous evolutionary rates decreased as disease progressed, supporting this scenario in addition to the first explanation. Thus, the decrease in the evolutionary rate at later stage of infection relates both to amino acid changing and non-changing nucleotide substitutions. Third, an increase of the viral fitness at later stages of infection may reduce further accumulation of mutations, finding a local fitness maximum in the rugged fitness landscape.

A correlation between the decline of diversity and the emergence of viruses using the CXCR4 coreceptor was reported in Ref. [13]. The surface expression of the HIV-1 coreceptors CCR5 and CXCR4 on CD4+ T-cells is differentially expressed on memory versus naïve T cells. A chemokine receptor CXCR4 is expressed on both memory and naive cells, although at greater levels on naive T-cells [14]–[17]. It has been reported that naïve T-cells are indeed infected and may act as an important viral reservoir in patients with CXCR4-using viruses [34]. Interestingly, our modeling revealed that the emergence of a fitter virus population, using CXCR4, resulted in a rapid increase both in divergence and diversity followed by the initial slope of linear increase of divergence and decline of diversity if the probability of mutations is a constant for all viruses.

Viral escape from neutralizing antibodies [8],[12],[35] and CD8+ T-cell responses [7],[10],[36],[37] suggest that, within a host, the HIV-1 sequence population is evolving in a dynamic environment of immune pressures. One of the selection forces controlling the evolution of *env* is escape from antibody neutralization. For instance, changes in N-linked glycosylation sites in *env* have been observed in viruses that escape antibody neutralization [12]. Also, as shown by an antibody neutralization assay, the virus population at a specific time point is neutralized more strongly with antibodies sampled at a later time point [8]. Interestingly, in Table 1 of reference [8], we observed that antibodies generated at later time points had a lower neutralizing capacity than those generated earlier during infection. For example, the maximum strength of neutralization against virus sampled at month 0 occurred with antibodies (plasma) sampled at month 12. Virus sampled 6 months later had a lower neutralizing titer with antibodies sampled at month 18, and the neutralization strength decreased as disease progressed. This observation suggests a weakening of the immune selection pressure during chronic infection. Furthermore, apparent decrease of CD8+ T-cell levels in HIV-1 chronic infection, as well as the exhaustion of CD8+ T-cells as mediated by the PD-1 molecule [38], both imply diminishing CD8+ T-cell responses over disease progression. Recent observations of selective depletion of high-avidity HIV-1 specific CD8+ T-cells after early HIV-1 infection also implies a lessening of CD8+ T-cell responses [39]. Thus, when the diversifying selection pressure on Env from the immune system weakens new escape mutations are not beneficial, and thus the probability to establish new mutations decreases. The immune pressure selects and removes all virus variants it can detect, while those escaping are an increasingly diverse set during chronic infection. When the immune system pressure fails in late stage disease this pressure to diversify is released and as a result, a relatively homogeneous sequence population is observed.

Previous studies have suggested an inverse relationship between disease progression and evolutionary rate based on the observation of enhanced viral escape under strong immune selection in slow progressors [2],[22]. Also, slower genetic diversification has been associated with rapid CD4+ T-cell decline [1],[20],[21],[40]. However, others have reported a positive relationship, suggesting that the evolutionary rate may be low in nonprogressors due to that immune selection may suppress emerging virus with potentially high fitness [23],[24]. To resolve these conflicting observations, we have shown that the rate of HIV-1 *env* evolution does not remain constant within a single infected individual, and thus simply correlating the average rate of evolution with disease progression may be misleading. Indeed, this may explain the contradictory results previously published. Thus, rather than using average rates, we show that the *dynamics* of the evolutionary rate reflects the rate of disease progression. In addition to the 13 out of 15 patients in Figure 7, 3 out of 6 rapid progressors in Ref. [23] show a decrease in the evolutionary rate when their CD4 cells rapidly deplete, while 3 non-progressors display a stable evolutionary rate.

Our estimates of the evolutionary rate were based on maximum likelihood trees calculated using realistic evolutionary substitution models [2],[30],[41]. However, these trees implicitly assume that no recombination has occurred, an assumption that may be violated by HIV-1 [42]–[45]. Detecting recombination among closely related HIV-1 sequences within a patient is difficult due to other evolutionary mechanisms causing a high degree of homoplasy (parallel and convergent mutations in different lineages), potentially misleading the analysis. Indeed, most of the patient sequence sets in this study suggest some degree of intra-population recombination strongly correlated to the degree of homoplasy in the dataset (r = 0.91). The recombination rates are estimated under the assumption that just a single mutation has caused each polymorphism within a group, and that there is no selection [46]. Because these assumptions are violated by HIV-1 *env* V3 we evaluated the potential recombination signal. It is well known that the *env* V3 region is under positive selection [47], which may lead to convergent evolution on some residues, explaining some of the homoplasy. In our data both synonymous/nonsynonymous mutation ratios and Tajima's D statistic [48] indicated departures from neutrality (Table 2). Importantly, previous studies have shown that recombination and selection rates may confound each other [49],[50]. Also, it is clear that the homoplasy increases as more sequences are investigated (Table 2). Thus, although difficult to exactly quantify, part of the detected recombination signal in our data could be explained by stochastic effects and convergent evolution. This potential recombination was also analyzed using SplitsTree [51]. Importantly, that analysis showed that if recombination had occurred, it mostly involved sequences collected closely in time. Therefore, the recombination in our data could only have affected our rate estimates mildly. Most important, and in agreement with previous publications using these data (e.g., [13],[19]), all our trees displayed a clear time-order of the samples (Figure 7), which would have been impossible if recombination had had a strong effect. Similarly, if ancestral (archival/latent) virus reemerged at later time points, we would have lost the time-order in the trees. In conclusion, neither recombination nor reemerging viruses could have had a strong effect on our rate estimates.

**Figure 10:**
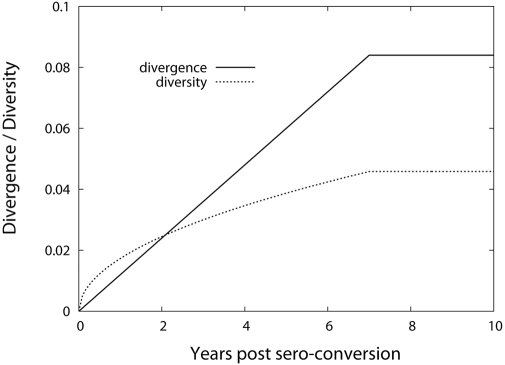
Dynamics of divergence and diversity from the model when the proportion of mutant offspring is set to zero after 7 years. Divergence and diversity dynamics calculated under an alternative model with a constant probability of mutation, 0.5, before time *τ* followed by zero after *τ*. Here *τ* is chosen as 7 years. Since evolution of total population stops at *τ*, divergence and diversity stay constants afterwards.

Williamson *et al.* [18] obtained maximum likelihood estimates for the mean divergence rate and the divergence stop time in each Shankarappa patient for the nonsynonymous and synonymous changes. They observed a strong relationship between the time of disease progression and the time of divergence stabilization only for nonsynonymous sites. The evolution profile in [18] corresponds to a constant evolutionary rate before time *τ* followed by zero evolutionary rate after *τ*. This kind of evolutionary profile can be imposed in our model by introducing the evolutionary profile depending on the time rather than the distance from the founder strain, *M*(*t*). Then Eq. (1) is modified as
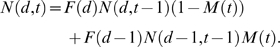
(18)The probability of mutation is a non-zero constant before *τ* and zero after *τ*, *M*(*t*) = *m* for *t*≤*τ* and *M*(*t*) = 0 for *t*>*τ*. We fix the fitness as a constant, *F*(*d*) = *f*. Figure 10 shows that not only divergence but also diversity saturates after *τ*. Since the evolution of the total population stops at *τ*, divergence and diversity stay constant afterwards. Hence, we can conclude that this alternative model, where the evolutionary profile depends on time, does not capture the decline of diversity at later stages of infection.

Similar to HIV-1, one study on intrahost sequence evolution in hepatitis C virus (HCV) reported that the diversity increased over time in non-progressors [52]. In contrast, progressors to end-stage liver disease showed that diversity in the hypervariable region I of E1/E2 *env* narrowed over time. We expect that the slowing down of the rate of HCV evolution also occurs as disease progresses, resulting in less diversity.

In conclusion, we observed that the evolutionary rate of the HIV-1 slows down in 13 of 15 patients from two independent previous studies [13],[22]. The rate of change in the evolutionary rate is correlated with the slope of CD4+ T-cell decline, dissolving previously reported conflicting observations of the relationships between the rate of HIV-1 evolution and disease progression. Our HIV-1 evolutionary model successfully captured the saturation of divergence and the decrease of diversity observed in the later stages of infection. In our model these effects are mostly attributed to a decrease in the proportion of offspring that are mutants in the population as the distance from the founder strain increases.

## Materials and Methods

### Samples and Sequence Data

We analyzed sequence data from two independent studies, the *env* C2-V5 region from nine patients [13] and the V3–V5 region from another six patients [22]. Briefly, sequences from the first nine patients were collected over their entire disease progression. The follow-up time varied between 6 to 12 years, at which time seven had developed AIDS and seven of the patients received antiretroviral treatment [13]. The other six patients were followed for 3 to 10 years [22]. Three of these patients received antiretroviral therapy 2–5 years after infection. All HIV-1 sequences were downloaded from the HIV database (GenBank Accession numbers AF137629-AF138163, AF138166-AF138263, AF138305-AF138703 and U35894-U36185).

### Reconstruction of Phylogenetic Trees

Sequences were aligned using Se-Al [53]. Trees were created using enhanced and parallelized versions of fastDNAml and Rates [54],[55], that fit a general-time-reversible substitution model (RevML) and site rate specific rates (RevRates) in an iterative way [41],[56]. Briefly, a candidate tree topology was created assuming uniform site rates and an initial random estimate of nucleotide frequencies and transition rates. RevML proceeds in a heuristic and piecewise way, starting from a small set of sequences and building up the tree topology and branch lengths while making placement decisions that maximize the tree likelihood score, similar to a stepwise addition algorithm. The resulting tree then constrains per-site rate optimization of tree likelihood as a function of global estimates of baseline nucleotide frequency and transition rates. These estimates are fit using the conjugate gradient algorithm in the RevRates program. A second RevML run was then performed using these estimates and in turn another rate estimation procedure refined from the second tree. A final tree was estimated using the twice-refined global and local site rates. Each of the trees in the refinement procedure was independently estimated from the global and site local rate parameters.

### Synonymous and Nonsynonymous Evolutionary Rates

Trees based on synonymous and non-synonymous substitutions, respectively, were inferred using HyPhy with optimized MG94xREV models [30]. This model uses a codon-based substitution model (MG94) that considers substitutions involving non-stop codons, augmented with a general-time-reversible nucleotide substitution model (REV) to include the heterogeneity in nucleotide frequencies and substitution rates [57]. The total number of changes per codon is decomposed into synonymous and nonsynonymous changes according to the universal coding table. Separate synonymous and nonsynonymous rates are then fitted to each branch of the tree. Prior to model fitting and tree reconstruction, alignments were codon corrected using the HyPhy SeqAlignment procedure (with the HIV-1 25% scoring matrix).

#### Calculation of Divergence and Diversity Dynamics

We calculated divergence by measuring the maximum likelihood tree distance from a sequence sampled at time *t* from a strain found at the earliest sample time point. Then we averaged all the pairwise tree distances between the sequences sampled at *t* and the sequence sampled at the earliest time point. The diversity was calculated from the data by averaging pairwise tree distances over all the sequences obtained at time *t*. Since the maximum likelihood tree was based on the nucleotide level, divergence and diversity were also calculated at a nucleotide level including coding and non-coding changes. The theoretical curves describing the evolution of divergence and diversity were computed assuming one time unit in the model corresponds to one month of evolutionary time.

### Estimating Population Polymorphism and Recombination

We used SITES [46] and SplitsTree [51] to investigate potential recombination signals in each patient set of sequences, PAUP* [58] to estimate the amount of homoplasy, SNAP [41] to estimate average synonymous/non-synonymous rates, and Tajima's D to estimate neutral evolution[48].

### Calculation of Evolutionary Rate

The rate of evolution was calculated in consecutive windows over a maximum likelihood (ML) tree from each patient, starting from the root. The distance to the root for all taxa in each window [*d*, *d*+Δ] was calculated from the tree (Figure 7A), and the resulting evolutionary rate was estimated as
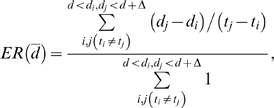
(19)where *d_i_*(*d_j_*) is the distance from the root of sequence *i*(*j*) at sampling time point *t_i_*(*t_j_*). Here *d̅* is the average distance from the root over all the sequences within the window, [d, *d*+Δ]. The window size was Δ = 0.09 for the Shankarappa data set and Δ = 0.02 for the Wolinsky data set. The average evolutionary rate over the entire sampling period from a patient was calculated as
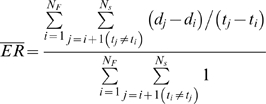
(20)by averaging the rate of evolution over the sequences in reference to the founder strains which are sampled at the earliest time point in each subject. Here, *N_F_* is the total number of founder strains and *N_s_* is the total number of sequences in a patient.
